# Advertisers, Communications, Etc.

**Published:** 1880-01

**Authors:** 


MARRIAGES, BIRTHS AND DEATHS.--We would like to make a record of all important events occurring in the families of our patrons and friends, and would, therefore, be obliged for items of births, marriages and deaths, as they may occur among them.

